# Supplementary material for: Divergent Directionality of Immune Cell-Specific Protein Expression between Bipolar Lithium Responders and Non-Responders Revealed by Enhanced Flow Cytometry
Source: Medicina (Kaunas). 2023 Jan 7;59(1):120. doi: 10.3390/medicina59010120 (PMC9860624; doi:10.3390/medicina59010120)
Supplement: Supplementary file 1 [file medicina-59-00120-s001.zip › medicina-2109334-supplementary.pdf]

Supplemental Table S1 Abbreviation of 28 proteins in the discovery analysis in the pilot study

Supplemental table XX

Function	Full name	Abbreviated name	Ref.
Apoptosis and anti-apoptosis	BCL2-Associated X Protein	BAK	
	B-cell lymphoma 2	Bcl-2	
	Bcl-2-related protein A1	Bcl-2 A1	
Calcium transport	Calcium-modulated protein	calmodulin	
Cell signaling	Glycogen synthase kinase 3 beta	GSK3β	
	Phosphorylated glycogen synthase kinase 3 alpha (Tyr279) beta (Tyr216)	Phospho-GSK3αβ	
	Phosphorylated glycogen synthase kinase 3 beta (Tyr216)	Phospho-GSK3β	
	inducible isoform nitric oxide synthase	iNOS	
	cAMP-specific 3',5'- cyclic phosphodiesterase 4B	PDEB4	
Circadian rhythm	Timeless	Timeless	
Metabolic enzymes	Phosphoglucomutase 1	PGM1	
	Tryptophan hydroxylase 1	THP1	
Inflammation	High mobility group box 1 protein	HMGB1	
	NACHT, LRR and PYD domains-containing protein 3	NLRP3	
	Tumor necrosis factor, alpha-induced protein 3	TNFAIP3	
	Myristoylated alanine-rich C-kinase substrate	MARCKS	
Kinase activity	Fyn	Fyn	
	Phosphorylated Fyn(Y530)/Yes(Y537)	p-Fyn/Yes	
	Protein kinase A catalytic subunit alpha	PKA C-α	
	Protein kinase C theta	PKC-δ	
	The mammalian target of rapamycin	mTor	
Neurotrophic factor	Brain derived neurotrophic factor	BDNF	
Receptors	Insulin receptor substrate 2	IRS2	
	Peroxisome proliferator-activated receptor gamma	PPAR-γ	
	Glucocorticoid receptor	NR3C1	
Transcription factor	Nuclear factor kappa B phospho-p65 (Ser536) subunit	p-RelA	
	phosphorylated cAMP response element-binding protein(Ser133)	phospho-CREB:	
	X-box binding protein 1	XBP1	

Supplemental Table S2. Protein levels between lithium responders and non-responders in lymphocytes before and after lithium treatment

	Before Lithium					After Lithium				
**Analytes**		**ITT-Responders (ITT-R)**		**ITT-Non-Responders (ITT-NR)**	**ITT-R vs. ITT-NR**		**CS-Responders (CS-R)**		**CS-Non-Responders (CS-NR)**	**CS-R vs. CS-NR**
	n	**Mean ± SD**	***n***	**Mean ± SD**	***p*-value**	***n***	**Mean ± SD**	***n***	**Mean ± SD**	***p*-value**
**BAK**	12	11.5 ± 2.9	11	11.2 ± 2.1	0.765	9	13.2±2.3	4	9.8±3.8	0.224
**BCL-2**	11	35.9 ± 8.9	11	39.7 ± 6.9	0.304	9	40.4±6.2	4	31.5±12.4	0.308
**BCL-2 A1**	12	1.2 ± 0.3	11	1.4 ± 0.2	0.150	9	1.3±0.1	4	1.2±0.1	0.118
**BDNF**	12	34.0 ± 7.4	10	37.8 ± 6.7	0.245	9	39.1±4.8	4	20.4±14.7	0.112
**Calmodulin**	12	31.9 ± 7.7	11	34.4 ± 5.7	0.407	9	36.3±5.2	4	29.3±11.8	0.385
**Fyn**	12	30.2 ± 6.4	11	33.0 ± 5.4	0.311	9	35.1±5.3	4	23.9±7.0	0.062
**GSK-3β**	12	19.5 ± 6.5	10	20.9 ± 7.9	0.660	9	24.5±3.2	4	18.6±5.3	0.150
**HMGB1**	11	22.6 ± 6.6	8	23.2 ± 8.7	0.880	8	23.5±4.8	4	20.2±7.0	0.495
**iNOS**	12	24.5 ± 7.5	11	24.5 ± 4.4	0.992	9	26.2±5.1	4	15.3±4.9	**0.019**
**IRS2**	12	24.1 ± 8.7	11	25.3 ± 5.6	0.716	9	27.0±5.6	4	24.8±9.0	0.707
**MARCKS**	12	1.28 ± 0.54	11	1.3 ± 0.4	0.853	9	1.4±0.4	4	1.1±0.3	0.259
**mTor**	12	21.1 ± 8.6	11	22.9 ± 6.4	0.605	9	22.5±3.7	4	17.8±6.5	0.304
**NR3C1**	8	6.7 ± 2.6	4	7.3 ± 2.2	0.687	9	31.4±4.8	4	28.6±14.0	0.751
**NLRP3**	12	26.0 ± 7.5	10	30.7 ± 5.7	0.136	9	9.3±2.5	4	4.6±2.1	**0.020**
**PDEB4**	10	27.3 ± 5.8	5	28.2 ± 9.1	0.837	9	32.1±4.5	3	25.3±9.	0.408
**phospho-CREB**	12	30.5 ± 6.6	11	30.8 ± 4.3	0.901	9	31.7±4.2	4	24.0±7.6	0.176
**phospho-Fyn Yes**	12	13.3 ± 6.4	11	12.5 ± 4.5	0.743	9	33.7±3.4	4	28.0±12.1	0.480
**phospho-GSK 3β**	12	4.1 ± 1.5	11	4.0 ± 0.8	0.865	9	16.4±4.5	4	11.4±2.4	**0.039**
**phospho-GSK 3αβ**	12	1.9 ± 0.6	11	2.3 ± 0.6	0.187	9	5.2±1.3	4	4.9±2.1	0.795
**Phospho-NFkB-P65**	12	14.6 ± 8.0	11	15.0 ± 6.0	0.887	9	2.0±0.6	4	2.0±0.4	0.958
**PGM1**	12	28.6 ± 6.6	11	31.0 ± 4.6	0.370	9	17.8±3.7	4	12.0±2.7	**0.023**
**PKA C-α**	12	23.9 ± 7.1	11	24.5 ± 5.7	0.843	9	28.7±4.1	4	13.1±8.9	**0.051**

**PKC-θ**	9	35.8 ± 8.7	5	33.8 ± 7.1	0.695	9	37.5±4.9	4	30.2±12.5	0.388
**PPAR-γ**	12	21.1 ± 5.6	11	21.9 ± 4.3	0.737	9	25.3±5.6	4	17.9±4.9	0.076
**Timeless**	12	1.42 ± 0.40	11	1.41 ± 0.12	0.9645	9	1.5±0.2	4	1.2±0.4	0.316
**TNFAIP3**	12	31.17 ± 6.64	11	33.74 ± 6.16	0.3756	9	34.6±4.7	4	25.0±7.0	0.093
**TPH1**	12	7.23 ± 2.47	11	7.13 ± 1.04	0.9106	9	8.5±1.7	4	5.8±1.4	**0.031**
**XBP1**	12	0.96 ± 0.20	11	1.11 ± 0.36	0.2262	9	1.1±0.1	4	1.0±0.1	0.393

BAK: BAX, BCL2-Associated X Protein; **BCL-2**: B-cell lymphoma 2; **BCL-2 A1**: Bcl-2-related protein A1; **BDNF**: brain-derived neurotrophic factor; **Calmodulin**: calcium-modulated protein; **GSK-3β**: glycogen synthase kinase 3 beta; **HMGB1**: High mobility group box 1 protein; **iNOS**: inducible isoform nitric oxide synthases; **IRS2**: Insulin receptor substrate 2; **MARCKS**: myristoylated alanine-rich C-kinase substrate; **mTor**: mammalian target of rapamycin; **NLRP3**: NACHT, LRR and PYD domains-containing protein 3; **NR3C1**: nuclear receptor subfamily 3, group C, member 1; **phospho CREB:** phosphorylated cAMP response element-binding protein (Ser133); **phospho Fyn /Yes**: phosphorylated Fyn(Y530)/Yes(Y537); **phospho GSK 3 α/β**: phosphorylated glycogen synthase kinase 3 alpha(Tyr279) beta(Tyr216); **phospho GSK 3β**: phospho-glycogen synthase kinase 3 beta(Tyr216)**; phospho NFKB-P65**: phosphorylated nuclear factor NF-kappa-B p65(Ser536) subunit; **PDEB4**: cAMP-specific 3',5'- cyclic phosphodiesterase 4B; PGM1: phosphoglucomutase 1; **PKA C-α**: protein kinase A catalytic subunit; **PKC-δ**: protein kinase C theta; **PPAR-γ**: peroxisome proliferator-activated receptor gamma; **TNFAIP3**: tumor necrosis factor, alpha-induced protein 3; **TPH1**: tryptophan hydroxylase 1; **XBP1**: X-box binding protein 1.

Supplemental Table S3. Comparison of protein levels in lymphocytes and monocytes at baseline between lithium completed responders and completed non-responders

	CD4^{+} Lymphocytes					Monocytes				
**Analytes**	**Completed Responders**		**Completed Non-Responders**		**RES vs. non-RES**	**Completed Responders**		**Completed Non-Responders**		**RES vs. non-RES**
	n	**Mean ± SD**	***n***	**Mean ± SD**	***p*-value**	***n***	**Mean ± SD**	***n***	**Mean ± SD**	***p*-value**
**BAK**	9	11.9±2.8	4	10.0±1.0	0.118	9	23.2±4.8	4	20.5±2.3	0.227
**Bcl-2**	8	37.3±9.3	4	36.2±7.9	0.861	8	22.1±4.7	4	22.9±4.7	0.826
**Bcl-2 A1**	9	1.2±0.2	4	1.4±0.1	0.324	9	1.4±0.3	4	1.5±0.2	0.743
**BDNF**	9	35.0±7.6	4	35.9±8.9	0.885	9	50.1±12.5	4	47.9±7.6	0.740

**Calmodulin**	9	33.6±7.5	4	31.0±6.5	0.583	9	48.4±12.1	4	41.6±9.2	0.352
**Fyn**	9	31.2±6.7	4	30.1±5.9	0.807	9	34.4±8.0	4	35.3±6.4	0.857
**GSK3β**	9	20.9±5.1	4	20.4±6.9	0.913	9	29.8±9.7	4	29.4±6.6	0.952
**HMGB1**	9	22.6±6.8	4	21.0±8.1	0.773	9	25.0±8.8	4	18.1±2.8	0.072
**iNOS**	9	24.2±7.6	4	21.7±3.5	0.473	9	26.5±13.0	4	24.0±5.1	0.657
**IRS2**	9	24.7±8.3	4	27.1±6.8	0.631	9	43.7±13.2	4	45.7±6.8	0.741
**mTor**	9	22.4±8.3	4	25.7±6.9	0.527	9	23.0±10.8	4	27.9±8.8	0.468
**MARCKS**	9	1.4±0.5	4	1.5±0.4	0.616	9	37.9±9.3	4	37.1±10.0	0.910
**NLRP3**	9	27.3±6.7	4	26.6±5.3	0.882	9	33.5±10.4	4	35.1±6.5	0.797
**NR3C1**	8	6.7±2.5	4	7.3±1.9	0.685	8	7.0±1.4	4	8.4±2.3	0.362
**PDEB4**	9	26.8±5.5	3	28.1±7.9	0.798	9	20.7±6.0	3	27.8±8.5	0.251
**PGM1**	9	29.9±6.5	4	29.4±5.1	0.883	9	41.5±10.5	4	39.8±6.7	0.758
**phospho-CREB**	9	30.7±6.6	4	30.7±4.1	1.000	9	46.3±9.2	4	46.8±5.9	0.927
**phospho-Fyn/Yes**	9	13.8±5.5	4	14.8±3.3	0.725	9	24.7±9.9	4	26.5±4.8	0.694
**phospho-GSKβ**	9	4.3±1.6	4	4.3±0.6	0.966	9	4.5±1.4	4	5.1±1.1	0.519
**phospho-GSK3αβ**	9	2.0±0.6	4	2.7±0.7	0.178	9	3.1±1.1	4	4.4±1.0	0.139
**Phospho-NFKB-P65**	9	16.2±7.5	4	15.2±4.5	0.800	9	9.7±5.1	4	8.7±2.8	0.668
**PKA C-α**	9	25.9±5.9	4	25.3±4.8	0.879	9	42.3±10.3	4	41.4±5.1	0.862
**PKC-θ**	9	35.8±8.2	4	33.8±6.2	0.674	9	5.2±1.9	4	6.0±2.6	0.639
**PPAR-γ**	9	21.4±5.7	4	22.1±5.2	0.842	9	40.3±10.9	4	39.9±7.8	0.936
**TNFAIP3**	9	31.6±6.6	4	29.3±3.2	0.462	9	22.8±9.0	4	19.5±2.4	0.372
**Timeless**	9	1.4±0.4	4	1.5±0.1	0.848	9	1.8±0.5	4	1.8±±0.1	0.800
**TPH1**	9	7.0±2.5	4	7.3±1.0	0.774	9	16.1±5.6	4	16.1±3.8	0.995
**XBP1**	9	1.0±0.2	4	1.3±0.4	0.283	9	1.3±0.3	4	1.6±0.6	0.363

BAK: BAX, BCL2-Associated X Protein; **BCL-2**: B-cell lymphoma 2; **BCL-2 A1**: Bcl-2-related protein A1; **BDNF**: brain-derived neurotrophic factor; **Calmodulin**: calcium-modulated protein; **GSK-3**β: glycogen synthase kinase 3 beta; **HMGB1**: High mobility group box 1 protein; **iNOS**: inducible isoform nitric oxide synthases; **IRS2**: Insulin receptor substrate 2; **MARCKS**: myristoylated alanine-rich C-kinase substrate; **mTor**: mammalian target of rapamycin; **NLRP3**: NACHT, LRR and PYD domains-containing

protein 3; **NR3C1**: nuclear receptor subfamily 3, group C, member 1; **phospho CREB:** phosphorylated cAMP response element-binding protein (Ser133); **phospho Fyn /Yes**: phosphorylated Fyn(Y530)/Yes(Y537); **phospho GSK 3 α/β**: phosphorylated glycogen synthase kinase 3 alpha(Tyr279) beta(Tyr216); **phospho GSK 3β**: phospho-glycogen synthase kinase 3 beta(Tyr216)**; phospho NFKB-P65**: phosphorylated nuclear factor NF-kappa-B p65(Ser536) subunit; **PDEB4**: cAMP-specific 3',5'- cyclic phosphodiesterase 4B; PGM1: phosphoglucomutase 1; **PKA C-α**: protein kinase A catalytic subunit; **PKC-δ**: protein kinase C theta; **PPAR-γ**: peroxisome proliferator-activated receptor gamma; **TNFAIP3**: tumor necrosis factor, alpha-induced protein 3; **TPH1**: tryptophan hydroxylase 1; **XBP1**: X-box binding protein 1.

Supplemental Table S4. Comparison of protein levels in CD4$^+$ lymphocytes between completed responder and non-responders before and after lithium

	**Completed-Responders**					**Completed-non-responders**				
**Analytes**	**Before lithium**		**After lithium**		**Before vs. After Lithium**	**Before lithium**		**After lithium**		**Before vs. After Lithium**
	n	Mean ± SD	*n*	Mean ± SD	*p*-value	***n***	**Mean ± SD**	***n***	**Mean ± SD**	***p*-value**
**BAK**	9	11.9±3.0	9	13.2±2.4	0.312	4	10.0±1.1	4	9.8±1.1	0.942
**Bcl-2**	8	37.3±9.9	8	39.7±6.7	0.574	4	36.2±9.1	4	31.5±9.1	0.599
**Bcl-2 A1**	9	1.2±0.3	9	1.3±0.1	0.394	4	1.4±0.2	4	1.2±0.2	0.166
**BDNF**	9	35.0±8.0	9	39.1±5.1	0.211	3	31.2±5.3	3	27.2±5.4	0.633
**Calmodulin**	9	33.6±8.0	9	36.3±5.5	0.417	4	31.0±7.4	4	29.3±7.5	0.837
**Fyn**	9	31.2±7.1	9	35.1±5.6	0.211	4	30.1±6.8	4	23.9±6.8	0.286
**GSK-3β**	9	20.9±5.4	9	24.5±3.4	0.117	4	20.4±7.9	4	18.6±8.0	0.726
**HMGB1**	8	22.4±7.7	8	23.5±5.2	0.732	4	21.0±9.3	4	20.2±9.3	0.899
**iNOS**	9	24.2±8.1	9	26.2±5.4	0.532	4	21.7±4.1	4	15.3±4.1	0.117
**IRS2**	9	24.7±8.8	9	27.0±5.9	0.517	4	27.1±7.8	4	24.8±7.8	0.729
**mTor**	9	1.4±0.6	9	1.4±0.4	0.781	4	25.7±8.0	4	17.8±8.0	0.198
**MARCKS**	9	22.4±8.8	9	22.5±3.9	0.975	4	1.5±0.5	4	1.1±0.5	0.248
**NLRP3**	9	27.3 ±7.1	9	31.4±5.1	0.177	3	26.6±6.4	3	22.2±6.4	0.600
**NR3C1**	8	6.7±2.7	8	8.9±2.5	0.107	4	7.3±2.2	4	4.6±2.2	0.150
**PDEB4**	9	26.8 ±5.9	9	32.1±4.8	***0.051***	3	29.4±10.7	3	25.3±10.7	0.667

**PGM1**	9	29.9±6.9	9	31.7±4.5	0.543	4	29.4±5.9	4	24.0±5.9	0.344
**phospho-CREB**	9	30.7±7.0	9	33.7±3.6	0.281	4	30.7±4.8	4	28.0±4.8	0.723
**phospho-Fyn/ Yes**	9	13.8±5.8	9	16.4±4.8	0.313	4	14.8±3.9	4	11.4±3.9	0.202
**phospho-GSK3β**	9	4.3±1.7	9	5.2±1.4	0.211	4	4.3±0.7	4	4.9±0.7	0.688
**phospho-GSK3αβ**	9	2.0±0.6	9	2.0±0.6	0.851	4	2.7±0.8	4	2.0±0.8	0.147
**Phospho-NFKB-P65**	9	16.2±8.0	9	17.8±4.0	0.584	4	15.2±5.2	4	12.0±5.2	0.338
**PKA C-α**	9	25.9±6.3	9	28.7±4.3	0.278	4	25.3±5.6	4	13.1±6.0	0.082
**PKC-θ**	9	35.8±8.7	9	37.5±5.2	0.613	4	33.8±7.1	4	30.2±7.1	0.672
**PPAR-γ**	9	21.4±6.1	9	25.3±5.9	0.184	4	22.1±6.0	4	17.9±6.0	0.346
**Timeless**	9	1.4±0.4	9	1.5±0.2	0.643	4	1.5±0.1	4	1.2±0.1	0.354
**TNFAIP3**	9	31.6±7.0	9	34.6±5.0	0.303	4	29.3±3.7	4	25.0±3.7	0.372
**TPH1**	9	7.0±2.6	9	8.5±1.8	0.177	4	7.3±1.2	4	5.8±1.2	0.170
**XBP1**	9	1.0±0.2	9	1.1±0.1	0.392	4	1.3±0.5	4	1.0±0.5	0.234

Abbreviations: BAK: BAX, BCL2-Associated X Protein; **BCL-2**: B-cell lymphoma 2; **BCL-2 A1**: Bcl-2-related protein A1; **BDNF**: brain-derived neurotrophic factor; **Calmodulin**: calcium-modulated protein; **GSK-3β**: glycogen synthase kinase 3 beta; **HMGB1**: High mobility group box 1 protein; **iNOS**: inducible isoform nitric oxide synthases; **IRS2**: Insulin receptor substrate 2; **MARCKS**: myristoylated alanine-rich C-kinase substrate; **mTor**: mammalian target of rapamycin; **NLRP3**: NACHT, LRR and PYD domains-containing protein 3; **NR3C1**: nuclear receptor subfamily 3, group C, member 1; **phospho CREB:** phosphorylated cAMP response element-binding protein (Ser133); **phospho Fyn /Yes**: phosphorylated Fyn(Y530)/Yes(Y537); **phospho GSK 3 α/β**: phosphorylated glycogen synthase kinase 3 alpha(Tyr279) beta(Tyr216); **phospho GSK 3β**: phospho-glycogen synthase kinase 3 beta(Tyr216)**; phospho NFKB-P65**: phosphorylated nuclear factor NF-kappa-B p65(Ser536) subunit; **PDEB4**: cAMP-specific 3',5'- cyclic phosphodiesterase 4B; PGM1: phosphoglucomutase 1; **PKA C-α**: protein kinase A catalytic subunit; **PKC-δ**: protein kinase C theta; **PPAR-γ**: peroxisome proliferator-activated receptor gamma; **TNFAIP3**: tumor necrosis factor, alpha-induced protein 3; **TPH1**: tryptophan hydroxylase 1; **XBP1**: X-box binding protein 1.
